# Exosomes derived from alcohol-treated hepatocytes horizontally transfer liver specific miRNA-122 and sensitize monocytes to LPS

**DOI:** 10.1038/srep09991

**Published:** 2015-05-14

**Authors:** Fatemeh Momen-Heravi, Shashi Bala, Karen Kodys, Gyongyi Szabo

**Affiliations:** 1 Department of Medicine, University of Massachusetts Medical School, Worcester, MA 01605, USA

## Abstract

Hepatocyte damage and inflammation in monocytes/macrophages are central to the pathogenesis of alcoholic hepatitis (AH). MicroRNAs (miRNAs) regulate all of these processes. MiRNA-122 is abundantly expressed in hepatocytes while monocytes/macrophages have low levels. The role of exosomes in AH and possible cross talk between hepatocyte-derived exosomes and immune cells is not explored yet. Here, we show that the number of exosomes significantly increases in the sera of healthy individuals after alcohol binge drinking and in mice after binge or chronic alcohol consumption. Exosomes isolated from sera after alcohol consumption or from *in vitro* ethanol-treated hepatocytes contained miRNA-122. Exosomes derived from ethanol-treated Huh7.5 cells were taken up by the recipients THP1 monocytes and horizontally transferred a mature form of liver-specific miRNA-122. *In vivo*, liver mononuclear cells and Kupffer cells from alcohol-fed mice had increased miRNA-122 levels. In monocytes, miRNA-122 transferred via exosomes inhibited the HO-1 pathway and sensitized to LPS stimulation and increased levels of pro-inflammatory cytokines. Finally, inflammatory effects of exosomes from ethanol-treated hepatocytes were prevented by using RNA interference via exosome-mediated delivery of a miRNA-122 inhibitor. These results demonstrate that first, exosomes mediate communication between hepatocytes and monocytes/macrophages and second, hepatocyte-derived miRNA-122 can reprogram monocytes inducing sensitization to LPS.

Exosomes are small vesicles (50–150 nm) that are shed from almost every cell type and carry a variety of bio-macromolecules including proteins, mRNA, microRNA (miRNA), and other non-coding RNAs^1^,^2^,^3^. Exosomes originate from multivesicular endosomes and are enriched in tetraspanin family (CD63, CD9, and CD 81). Exosomes are released by a variety of cell types into the cellular microenvironment which makes them detectable in biofluids such as serum, plasma, and saliva^2^,^3^,^4^,^5^. Exosomes are gaining attention as new biomarkers of diseases. The wide variety of RNAs, which are packaged into the exosomes, can yield a molecular signature that is informative about physiological status and disease condition^2^,^5^.

Recent data suggest a novel role for exosomes as natural conveyors of information between cells and across various tissues through horizontal transfer of macromolecules. The first report of horizontal transfer of nucleic acids between cells was by Valadi *et al*. (2007), demonstrating that exosomes derived from mast cells can transfer RNA to other mouse and human mast cells^6^. Another study demonstrated horizontal transfer of fluorescently labeled glioblastoma-derived exosomes to human brain microvascular endothelial cells^7^. The role of exosomes in alcoholic liver disease is yet to be explored.

Exosomes play a role in the pathogenesis of different diseases and can also affect cells by transfer of lipids, protein, or genetic cargos. Recent investigations have shown their role in immune response^8^, angiogenesis^9^, thrombosis^10^, tumor invasion, and proliferation^11^. The quantity of circulating exosomes is elevated in various diseases including malaria^12^, melanoma^13^, ovarian cancer^14^, renal cancer^15^, diabetes mellitus^16^, and chronic renal failure^17^. Exosomes have the capacity to functionally transfer different bio-macromolecules in various pathological conditions. For example, in leishmaniasis, exosomes mediate the transfer of virulence factor to the host cells^18^. In diabetes, exosomes mediate transfer of auto-antigens and play a role in autoimmune trigger in diabetic mice^19^. A recent study showed an increased number of exosomes in different liver diseases^20^, however, the role of exosomes in alcoholic hepatitis (AH) remains unclear.

It has been shown that gut microbiome-derived lipopolysaccharides (LPS) and pro-inflammatory cytokines such as TNFα and IL-1β play pivotal roles in alcoholic hepatitis (AH) in animal models, indicating that alcohol-related inflammation contributes to the development of AH^21^. Functional studies on AH demonstrated the role of inflammatory mediators, pro-inflammatory cytokines, anti-inflammatory cytokines, and hepatoprotective cytokines in the pathogenesis of AH^21^. The identification of multiple inflammatory inducers in the pathogenesis of AH, including alcohol, its metabolites, and subsequent cellular changes have been well documented^22^. We recently showed that hepatocyte damage was a prerequisite of alcohol-induced liver inflammation^23^. However, the role of exosomes in possible cross talk between hepatocytes and immune cells is yet to be explored.

Liver resident cells including hepatocytes, hepatic stellate cells, Kupffer cells, sinusoidal endothelial cells, and recruited immune cells participate in the pathogenesis of alcoholic liver disease. Immune cells including monocytes, macrophages, T-cells, and dendritic cells are affected by alcohol and release pro-inflammatory cytokines, chemokines, lipid messengers, and reactive oxygen species that further augment cell damage^24^. Our group previously showed the presence of circulating exosomes in a mouse model of AH^25^^25^. These circulating exosomes were rich in miRNA-122, which is a liver-specific miRNA and abundant in the hepatocytes^26^. MiRNA-122 is one of the first identified tissue specific miRNAs and is highly enriched in the liver, but absent or expressed at very low abundance in other tissues and other cell types like immune cells, where its function is unknown^26^. We hypothesized that exosomes derived from ethanol-treated hepatocytes can convey messages to the monocytes and modulate their immune function.

In this study, we show that the total number of exosomes is significantly increased in the circulation after binge alcohol consumption in healthy human subjects and in mice, as well as after chronic alcohol consumption in mice. We also demonstrate that exosomes isolated from sera of healthy subjects after alcohol binge drinking were enriched in miRNA-122, demonstrating a rapid increase after alcohol consumption. We found that sorting of miRNAs into exosomes is a specific process and exosomes derived from ethanol-treated hepatocytes carry different cargos compared to the control exosomes. Mechanistically, our results revealed that ethanol treatment in hepatocytes increases the number of exosomes in a dose-dependent manner. These exosomes contained miRNA-122 and horizontally transferred miRNA-122 to monocytes. Importantly, exosome-modulated miRNA-122 transferred sensitized monocytes to inflammatory responses. Finally, we introduced a miRNA-122 inhibitor to THP1 cells via exosome-mediated delivery to block the effects of exosomes derived from ethanol-treated hepatocytes on the monocytes.

## Result

### Acute alcohol binge increases the number of circulating exosomes in healthy human subjects and in mice

**Figure 1:**
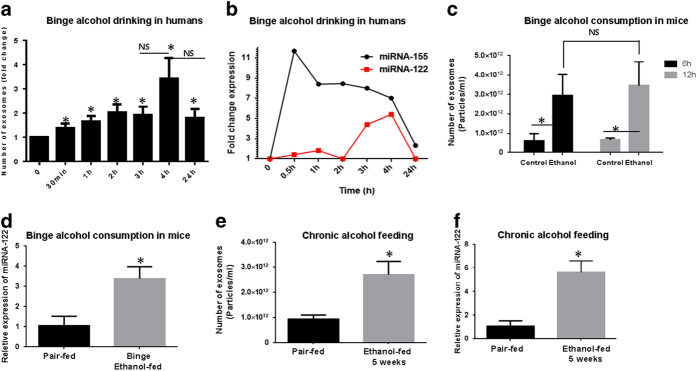
Number of exosomes in sera of human subjects and mice after alcohol consumption. **A**) Number of exosomes after binge alcohol drinking in healthy volunteer human subjects (n = 11) was measured at various time points (30 minutes, 1 h, 2 h, 3 h, 4 h, 24 h). The measurements were done using a Nanoparticle Tracking Analysis (NTA) system in triplicate and data presented as fold change of exosome number. **B**) Exosomes isolated from sera of healthy individual after binge alcohol drinking as described in the method section. TaqMan® miRNA assay was used to identify levels of miRNA-122 and miRNA-155. **C**) The animals (n = 6) received 5 g/kg 50% (v/v) alcohol diluted in water via oral gavage. Blood was collected from animals and serum was separated from the whole blood. The total number of exosomes in sera of mice 6 h and 12 h after binge alcohol drinking was counted using an NTA system and data presented as particles/ml. **D**) Exosomes isolated from sera of binge alcohol-fed mice as described in the method section. Using TaqMan® miRNA assay, the level of miRNA-122 was identified. **E**) For chronic alcohol consumption model, the animals (n = 6) received 5% (v/v) ethanol (36% ethanol-derived calories) containing Lieber-DeCarli diet (EtOH) for 5 weeks and control animals received pair-fed diet (PF) with an identical amount of calories where the alcohol-derived calories were substituted with dextran-maltose. Blood was collected from animals and serum was separated from the whole blood. The exosomes were counted using an NTA system and data presented as particles/ml. **F**) Exosomes isolated from sera of 5-week alcohol fed mice and level of miRNA-122 was determined using TaqMan® miRNA assay. The results represent three independent experiments. (*indicates p < 0.05 versus control conditions)

Binge alcohol drinking is associated with deleterious health effects. Recently we showed increased circulating endotoxin and bacterial 16S RNA in healthy individuals after binge drinking^27^. To identify the change in the number of exosomes after binge alcohol drinking, we measured the number of exosomes in the sera of healthy individuals (n = 7) before and after binge alcohol drinking at various time points (30 minutes-24 h) using a Nanoparticle Tracking Analysis (NTA) system. The total number of exosomes increased significantly after *in vivo* alcohol binge consumption in human subjects, as early as 30 minutes after drinking (p < 0.05), and remained significantly elevated at later time points (1 h, 2 h, 3 h, 4 h, and 24 h) (Fig. 1A). In exosomes isolated from sera of those individuals, the level of miRNA-122, a liver-specific miRNA, was increased to the maximum level after 4 h of alcohol consumption compared to the pre-alcohol levels. As our group previously showed increased level of miRNA-155 in the sera of chronic-alcohol fed mice^25^, we measured miRNA-155 in those samples. Interestingly, the kinetics of miRNA-155 peaked at 30 minutes after alcohol consumption, indicating different kinetics of miRNA packaging compared to the miRNA-122 in the exosomes after alcohol consumption (Fig. 1B). No changes in the number of exosomes or miRNA-122 content of exosomes were seen in the control individuals who received same volume of orange/strawberry juice at various time points (data not shown).

In a mouse model of binge alcohol consumption, the total number of exosomes increased significantly 6 h and 12 h after alcohol consumption in sera of mice (p < 0.05) (Fig. 1C). Consistent with the human data, the relative expression levels of miRNA-122 in exosomes isolated from binge alcohol fed-mice (12 h) was significantly increased compared to saline-fed animals (p < 0.05) (Fig. 1D). Because we previously observed liver damage and cell death in the hepatocytes in chronic alcohol-fed mice^23^, we next evaluated the number of circulating exosomes and the level of miRNA-122 in exosomes from the sera of chronic alcohol-fed mice. After 5-weeks of chronic alcohol feeding in mice, the number of circulating exosomes was significantly higher compared to the pair-fed controls (p < 0.05) (Fig. 1E). The relative levels of miRNA-122 in the exosomes were also significantly increased after 5 weeks of chronic alcohol feeding compared to the pair-fed mice (p < 0.05) (Fig. 1F).

### Alcohol induces exosome production in hepatocytes

**Figure 2:**
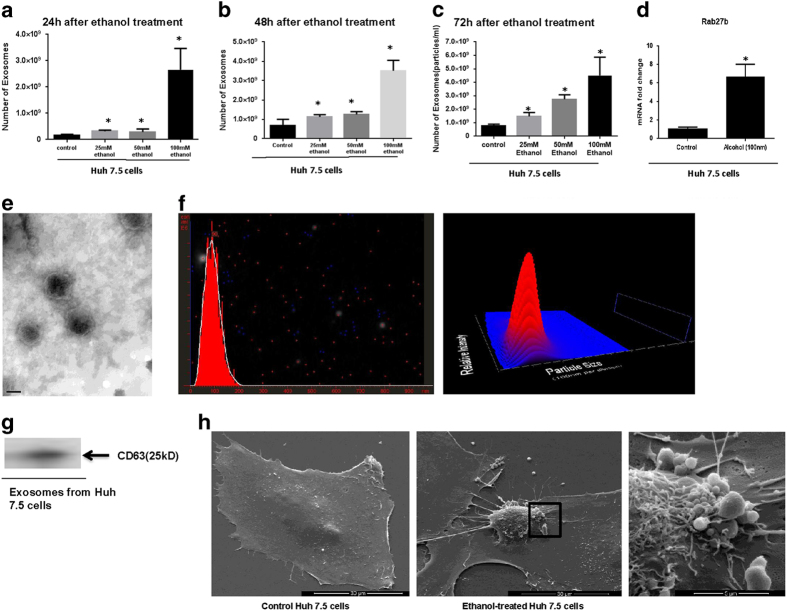
Kinetics of exosome production in the presence of ethanol and exosome characterization in Huh7.5 hepatoma cell line. **A**) Different doses of ethanol were added to Huh 7.5 cells for 24 h. Total number of exosomes was measured 24 h after ethanol treatment with different dosage including 25 mM, 50 mM, and 100 mM by an NTA system. Data presented as particles/ml. **B**) Different doses of ethanol were added to Huh 7.5 cells for 48 h.Total number of exosomes was measured 48 h after ethanol treatment with different dosage including 25 mM, 50 mM, and 100 mM by an NTA system. **C**) Different doses of ethanol were added to Huh 7.5 cells for 72 h.Total number of exosomes measured 72 h after ethanol treatment with different dosage including 25 mM, 50 mM, and 100 mM by an NTA system. **D**) The expression level of RAB 27b mRNA was identified in the hepatocytes after alcohol treatment using a quantitative real-time PCR. 18S was used as internal control for qPCR analysis. **E**) Exosomes derived from Huh 7.5 cells were negatively stained with 2% uracyl acetate after removing the extra moisture and visualized using an electron microscopy. **F**) The mean diameter (nm) of the exosomes derived from Huh7.5 cells was identified by a Nanoparticle Tracking Analysis (NTA). **G**) The presence of exosomal marker CD63 in exosomes derived from Huh 7.5 cells were identified by western blotting as shown in the single cropped blot. **H**) SEM of Huh 7.5 cells treated with alcohol compared to control Huh 7.5 cells (2 kX). The area in the box is magnified in the right picture (10 kX). (*indicates p < 0.05 versus control conditions) The quantitative results represent three independent experiments.

Because alcohol increased the number of circulating exosomes and miRNA-122 is the most abundant and specific miRNA in hepatocytes^26^, we next investigated the effect of ethanol treatment on exosome production in hepatocytes. We treated Huh7.5 cells with different concentrations of ethanol (25 mM [moderate dose], 50 mM [heavy dose], 100 mM [binge/chronic drinking]) and measured the changes in exosome production by a Nanoparticle Tracking Analyzing (NTA) system at 24 h, 48 h, and 72 h after ethanol treatments. The total number of exosomes increased significantly after ethanol treatment for 24 h, 48 h, and 72 h at various concentrations (25 mM, 50 mM, 100 mM) (p < 0.05) (Fig. 2A,B,C). The increase in the number of exosomes was time and dose dependent. The mRNA expression level of Rab 27b, the protein that plays a role in late endosomal formation^28^, was also significantly increased after ethanol treatment (p < 0.05), indicating activation of exosome biogenesis pathways by alcohol treatment (Fig. 2D).

### Characterization of hepatocyte (Huh7.5 cell) derived exosomes and their cargo

Exosomes derived from Huh7.5 cells had the reported size range of 50–150 nm and showed the previously described mushroom shape morphology on transmission electron micrographs (Fig. 2E)^29^. Exosomes had a mean diameter of 90 nm (Fig. 2F) and expressed the exosomal marker, CD63, identified by western blot (Fig. 2G). Consistent with the increase in the count of exosomes after ethanol treatment, Huh 7.5 cells treated with ethanol showed an increased number of shedding exosomes/microvesicles compared to control Huh 7.5 cells based on the scanning electron microscopy (SEM) graph. (Fig. 2H).

**Figure 3:**
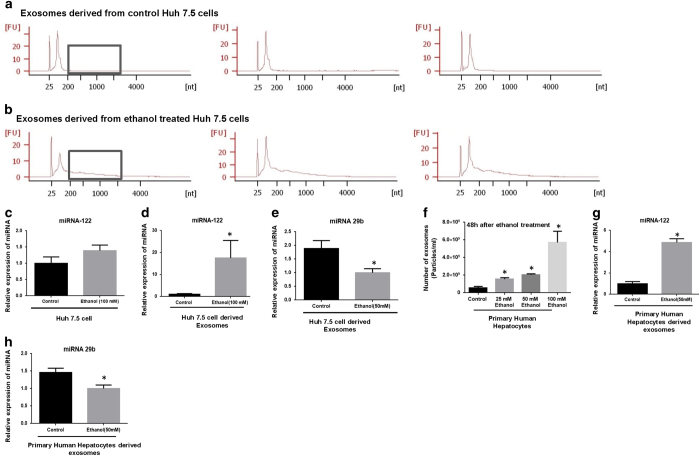
Exosome derived from hepatocytes treated with ethanol carry different signatures of small RNA and miRNA. **A&B**) Agilent bioanalyzer small RNA kit was used to characterize the distribution of packaged RNA into the exosomes. The first row (**A**) shows small RNA profile in the control exosomes derived from Huh7.5 cells in three different samples. The second row (**B**) shows small RNA profile in the exosomes derived from ethanol treated Huh7.5 cells in three different samples. Difference in abundance of long non-coding RNAs in exosomes derived from ethanol-treated cells compared to control exosomes is annotated by gray boxes in the graphs. (**C**) Using TaqMan® miRNA assay, levels of miRNA-122 were identified in the Huh 7.5 cells treated with ethanol (100 mM) and control Huh 7.5 cells. (**D**) Using TaqMan® miRNA assay, levels of miRNA-122 were identified in the exosomes derived from ethanol-treated Huh7.5 cells (100 mM) and control exosomes. (**E**) The levels of miRNA-29b were identified in the exosomes derived from ethanol-treated Huh7.5 cells (100 mM) and control exosomes, using TaqMan® miRNA assay. (**F**) Total number of exosomes was measured 48 h after ethanol treatment of primary human hepatocytes with different dosage including 25 mM, 50 mM, and 100 mM, using an NTA system. The total number presented as particles/ml. (**G**) The levels of miRNA-122 was measured in primary human hepatocytes after ethanol treatment (50 mM for 48 h), using TaqMan® miRNA assay. (**H**) The levels of miRNA-29b were measured in the exosomes derived from primary human hepatocytes after ethanol treatment (50 mM for 48 h), using TaqMan® miRNA assay. Results are representative of three independent experiments. (*indicates p < 0.05 versus control conditions)

Using small RNA Agilent bioanalyzer chip, we found that the profile of small RNA cargo in the exosomes derived from ethanol (100 mM) treated Huh7.5 cells and control cells are different, indicated by more abundance of long non-coding RNAs in exosomes derived from ethanol treated cells, compared to control exosomes (Fig. 3A,B). Although Huh 7.5 cells treated with ethanol showed slight increase in miRNA-122 levels, exosomes derived from ethanol-treated Huh7.5 cells, showed significantly elevated levels of miRNA-122 (p < 0.05) (Fig. 3C,D). Exosomes derived from ethanol treated Huh 7.5 cells showed decreased levels of miRNA-29b, a tumor suppressor miRNA^30^, compared to the exosomes derived from control cells (p < 0.05) (Fig. 3E). These data indicate that sorting of miRNA into the exosomes is a regulated process and it is specific for each miRNA.

### Primary human hepatocytes release exosomes after ethanol treatment

Next, we investigated the effect of ethanol treatment on exosome production in human hepatocytes. Primary human hepatocytes were tested with different concentrations of ethanol (25 mM, 50 mM, 100 mM) and the changes in exosome production were measured by Nanoparticle Tracking Analyzing (NTA). The total number of exosomes increased significantly after ethanol treatment for 48 h at various concentrations (25 mM, 50 mM, 100 mM) (p < 0.05) (Fig. 3F). Similar to Huh7.5 cells, the same profile of elevated levels of miRNA-122 and decreased levels of miRNA-29b were observed in exosomes derived from primary human hepatocytes after alcohol treatment (Fig. 3G,H). These data show that the sorting of miRNA into exosomes is a specific and regulated process and it is specific to the cell status.

### Exosomes derived from ethanol-treated hepatocytes transfer mature form of miRNA-122 to monocytes

**Figure 4:**
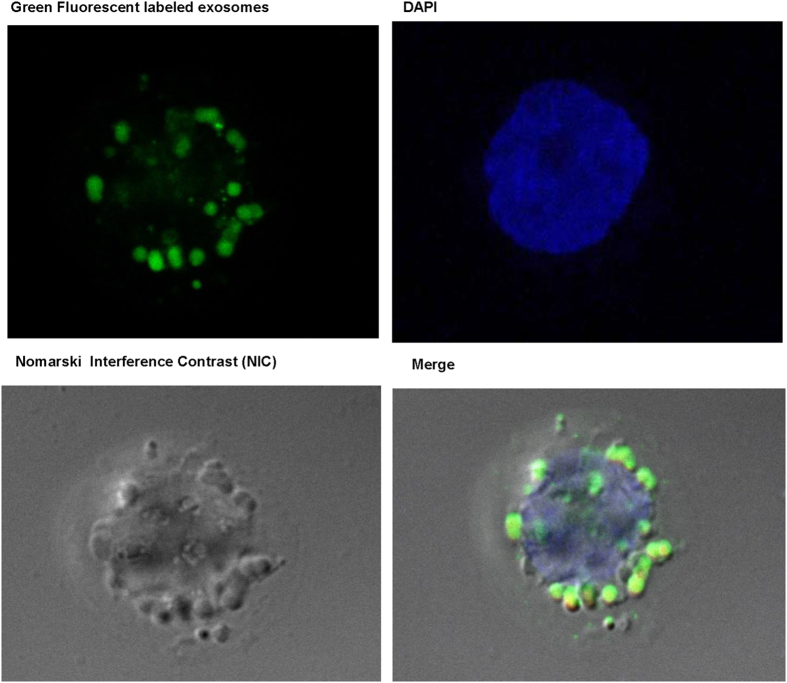
Confocal microscopy- Green Fluorescent labeled exosomes were taken up by THP1 monocytes as early as 6 h after Treatment. Exosomes were labeled with green fluorescent dye (PKH67) and co-cultured with THP1 cells (6 h). Nuclei were stained with DAPI and Nomarski Interference Contrast (NIC) was used for locating the cytoplasm. Exosomes were taken up by the THP1 cells indicated by the presence of green fluorescently labeled exosomes in the cytoplasm of the THP1 monocytes after merging the images.

Our results indicated that the mature form of miRNA-122, a liver-specific miRNA, was increased in the exosomes derived from ethanol-treated Huh7.5 cells and primary human hepatocytes (Fig. 3). Therefore, to investigate possible cross talk between ethanol-treated hepatocytes and immune cells, we treated THP1 human monocytes with ethanol-treated Huh7.5 cell-derived exosomes. After a 6-hour co-culture of PKH2 green fluorescently labeled hepatocyte-derived exosomes with THP-1 monocytes, confocal microscopy showed that exosomes were taken up by THP1 cells (Fig. 4).

**Figure 5:**
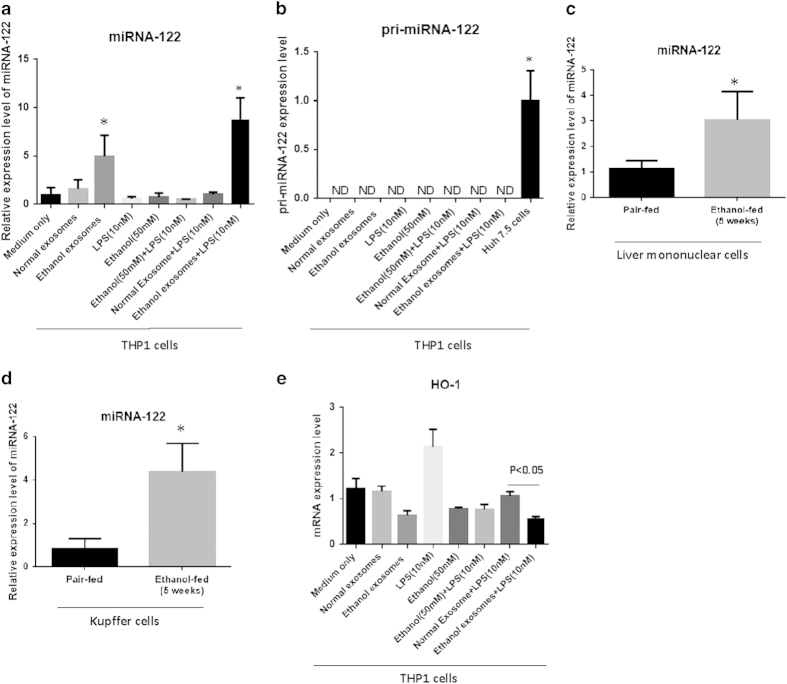
Ethanol-treated Huh7.5 cell derived exosomes horizontally transfer mature form of miRNA-122 to the THP1 cells. **A)** THP1 cells were treated with exosomes for 8 h and after that exosomes were washed off and media replaced. MiRNA-122 levels were identified in THP1 cells using a TaqMan® miRNA assay. RNU-48 was used to normalize the Ct values between the samples. **B**) TaqMan® Pri-miRNA Assays was used to quantify pri-miRNA-122 in THP1 cells. Extracted RNA from Huh7.5 cells served as a positive control. GAPDH was used as an internal control for gene expression analysis. **C**) miRNA-122 levels were compared between ethanol-feed mice and pair-fed mice in isolated liver mononuclear cells (MNCs) (n = 8). snoRNA202 was used to normalize the Ct values between the samples. **D**) miRNA-122 levels were compared between ethanol-feed mice and pair-fed mice in isolated Kupffer cells (n = 8). snoRNA202 was used to normalize the Ct values between the samples. **E**) Levels of Heme oxygenase 1(HO-1) mRNA, reciprocal target of miRNA-122, were measured in different experimental groups using quantitative real-time PCR. 18S was used as an internal control for quantitative real-time PCR analysis. The results represent three independent experiments. (*indicates p < 0.05 versus control conditions)

As previously reported, miRNA-122 represents 70–80% of the total miRNA in hepatocytes^26^. In monocytes, we found only a low copy number of baseline miRNA-122. Next, we showed that exosomes can mediate successful horizontal transfer of their miRNA-122 cargo to the THP1 cells, indicated by a 5–8 fold increase in the level of the mature form of miRNA-122 in monocytes treated with exosomes derived from ethanol-treated Huh7.5 cells (Fig. 5A). To rule out the induction of endogenous expression of miRNA-122 in THP1 cells, we evaluated the expression level of pri-miRNA-122 and found pri-miRNA-122 expression exclusively in hepatocytes but not in monocytes, even after co-culture with exosomes from ethanol-treated hepatocytes (Fig. 5B). Consistent with *in vitro* findings, liver mononuclear cells (MNCs) and Kupffer cells isolated from chronic alcohol-fed mice showed increased levels of MiRNA-122 compared to pair-fed mice (Fig. 5C,D).

### MiR-122 transferred by hepatocyte-derived exosomes is functional and modulates monocyte function

Although miRNA-122 and pri-miRNA-122 are almost absent in the THP-1 cells, HO-1, a miRNA-122 direct reciprocal target is present in the monocytes 31,32,33,34. Importantly, we observed a significant decrease in the level of HO-1 expression, a target of miRNA-122^31^, after treatment with exosomes from alcohol-treated hepatocytes into THP1 cells (Fig. 5E). HO-1 has inhibitory effects on cytokine- and reactive oxygen species- mediated cell damage^32^,^33^,^34^.

**Figure 6:**
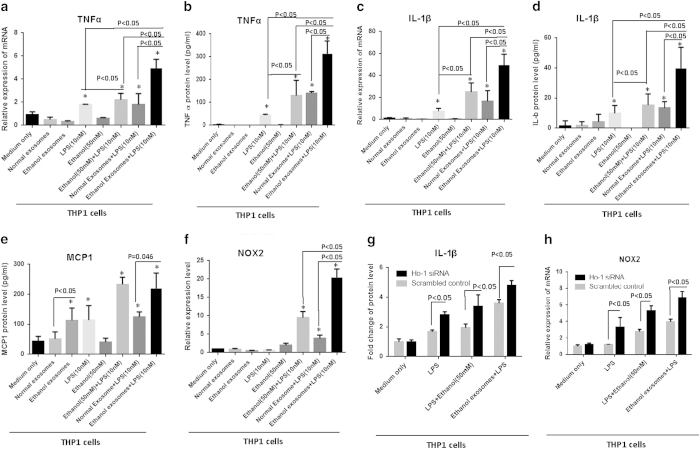
Immunomodulatory effects of ethanol-treated hepatocytes on human THP1 monocytes. Huh7.5 cells were treated with 100 mM ethanol for 48 h and exosomes were isolated with combination of filtration and ExoQuick-TC. Isolated exosomes from ethanol treated cells and non-ethanol treated cells (normal exosomes) were co-cultured with THP1 human monocytes for 8 h. After 8 h, exosomes were washed off and media replaced. 16 h later, RNA and cell supernatant were harvested and analyzed for target measurements. In the groups containing LPS, 10 nM was added to the THP1 cells 6 h before harvesting. **A**) The levels of TNFα mRNA expression were measured using quantitative real-time PCR (qPCR). 18S was used to normalize the Ct values between the samples. **B**) The levels of TNFα protein in the supernatant were measured by ELISA. **C**) The levels of IL-1β mRNA expression in THP1 cells were measured using qPCR. 18S was used to normalize the Ct values between the samples. **D**) The levels of IL-1β in the supernatant were measured by ELISA. **E**) The levels of MCP1 in the supernatant were measured by ELISA. **F**) The level of Nox2 mRNA expression in the THP1 cells were measured with qPCR. 18S was used to normalize the Ct values between the samples. **G**) HO-1 siRNA and scrambled siRNA control were introduced to THP-1 cells by electroporation. 2 × 10^5^ cells were re-suspended in 150 μl complete RPMI media and 150 μl Gene Pulser®Electroporation buffer for 5 min on ice before being electroporated. Electroporation was done at 300 kV and 1500 μF. Following electroporation, cells were kept on ice for 10 min then cultured in media for 48 hours. 10 nM LPS was added to the THP1 cells in the pertinent groups 6 h before harvesting. Ethanol exosome was added 24 h before harvesting. After 48 h supernatants were collected and levels of IL-1β were quantified by an ELISA. **H**) After knockdown of HO-1 by introducing siRNA to the THP1 cells as described in the previous section, RNA was extracted and expression levels of Nox2 mRNA were measured using qPCR. 18S was used to normalize the Ct values between the samples. The results represent three independent experiments. (*indicates p < 0.05 versus control condition)

In the presence of LPS, there was a statistically significant increase in TNFα and IL-1β both at the mRNA and secreted protein levels in THP1 cells after treatment with exosomes derived from ethanol-treated hepatocytes, compared to normal exosomes (p < 0.05) (Fig. 6A,B,C,D). LPS and LPS + ethanol induced a significant increase in TNFα and IL-1β mRNA and secreted protein levels versus control cells. The presence of exosomes derived from ethanol-treated hepatocytes (ethanol exosomes), induced a statistically significant pro-inflammatory response compared to the normal exosomes on the production of TNFα and IL-1β in both mRNA and protein levels (p < 0.05). Our data suggest that exosomes derived from ethanol-treated hepatocytes have a sensitizing effect on THP1 monocytes to an LPS challenge. MCP1 protein induction showed the same pattern, but did not reach statistical significance (Fig. 6E). Moreover, our results indicate that, in our *in vitro* setting, ethanol per se does not induce significant the pro-inflammatory profile and cytokine production in monocytes, but through its effect on enhancing number of exosomes in hepatocytes which harbor miRNA-122, indirectly induces the pro-inflammatory profile in monocytes.

Nox2, an HO-1 regulated gene of the cellular machinery producing reactive oxygen species is mainly expressed in immune cells and plays a pivotal role in host defense^35^,^36^. Consistent with the sensitizing effect of exosomes derived from ethanol-treated Huh7.5 cells, we found a statistically significant increase in Nox2 levels in THP1 cells after treatment with ethanol exosomes in the presence of LPS stimulation, compared to control groups (p < 0.05) (Fig. 6F).

Next, to test the hypothesis that the monocyte inflammatory phenotype was due to the decrease in the miRNA-122 targeted HO-1 expression, we introduced HO-1 siRNA via electroporation to knockdown HO-1 expression in THP1 monocytes. After 48 h, we challenged cells with 10 nM LPS, and measured IL-1β production. Similar to the THP1 cell phenotype after treatment with exosomes derived from ethanol-treated hepatocytes, a knockdown of HO-1 in THP1 cells resulted in a statistically significant increase in IL-1β protein production after the LPS and ethanol exosomes + LPS challenges (Fig. 6G). Consistently, after a knockdown of HO-1 we observed a significant increase in Nox2 levels in the presence of LPS and LPS + ethanol exosomes (Fig. 6H). Altogether, our data showed the successful horizontal transfer of liver specific miRNA-122 after ethanol treatment via exosomes, which is functional and modifies THP1 responses to LPS through modulating the HO-1 pathway.

**Figure 7:**
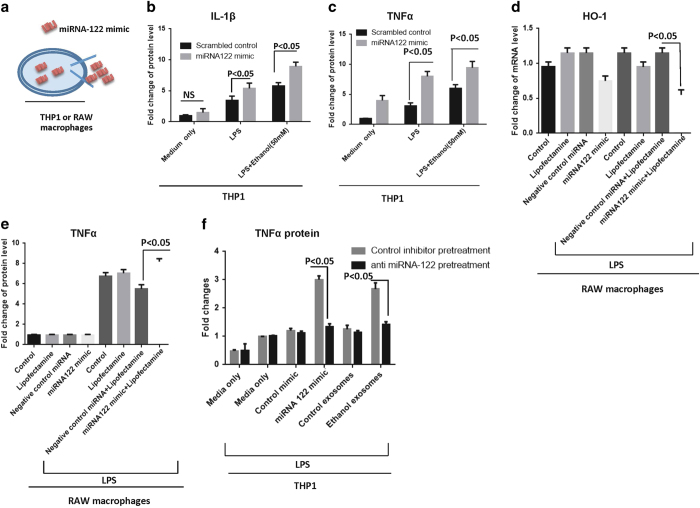
Simulation experiments to confirm production of pro- inflammatory cytokines due to miRNA-122 transfer and preventing inflammatory effects of ethanol exosomes using exosome-mediated delivery of RNAi. **A**) To confirm that horizontal transfer of miRNA-122 is inducing pro-inflammatory phenotype in monocytes, we designed “simulation experiments” in which miRNA-122 mimic was introduced to THP1 cells and RAW macrophages via electroporation and transfection reagents, respectively. **B**) MiRNA-122 mimic and control mimic were introduced to the THP1 cells with electroporation (300 kV and 1500 μF), after 18 h, 10 nM LPS was added for 6 h, supernatants were collected for IL-1β ELISA analysis. The results represent three independent experiments and expressed as IL-1β protein levels fold change. **C**) MiRNA-122 mimic and control mimic were introduced to the cells with electroporation (300 kV and 1500 μF), after 18 h, 10 nM LPS was added for 6 h and after that supernatants were collected for TNFα ELISA analysis. The results represent three independent experiments expressed as TNFα protein level fold change. **D**) miRNA-122 and negative control were introduced to the RAW macrophages with Lipofectamine® RNAiMAX transfection reagent and LPS (10 nM) was added 6 h before readings. After 48 h, HO-1 mRNA levels were measured by quantitative real-time PCR. 18S was used to normalize the Ct values between the samples. **E**) miRNA-122 and negative control were introduced to the RAW macrophages with Lipofectamine® RNAiMAX transfection reagent and LPS (10 nM) was added 6 h before readings. After 48 h, TNFα protein levels were measured by quantitative real-time PCR. **F**) miRNA-122 inhibitor was loaded into THP1-derived exosomes as described in the method section. Exosomes were added to naïve THP1 cells for 12 h; after 12 h exosomes were washed off and media was replaced. Exosomes derived from ethanol-treated Huh7.5 cells were added for 8 h. TNFα ELISA were done after 24 h and 6 h before readout 10 nM LPS was added to pertinent groups. MiRNA-122 mimic was electroporated to the THP1 cells as a positive control. The results represent three independent experiments.

To further confirm that the introduction of miR-122 to THP1 cells can modulate HO-1 and sensitize the monocytes to the LPS, we designed “simulation experiments” and introduced miRNA-122 to human THP1 monocytes and RAW murine macrophages via electroporation and transfection reagents, respectively (Fig. 7A). As shown in Fig. 7B,C, transfection of miRNA-122 mimic resulted in significantly higher protein levels of pro-inflammatory cytokines IL-1β and TNFα in the presence of LPS stimulation in THP1 monocytes (p < 0.05). This mimicked the sensitizing effect that we observed in the presence of exosomes derived from ethanol-treated hepatocytes, which abundantly harbor the mature form of miRNA-122.

The RAW 264.7 macrophages transfection of a miRNA-122 mimic using Lipofectamine, significantly decreased the expression level of HO-1 mRNA (p < 0.05) and increased TNFα protein level (p < 0.05), compared to the control group transfected with negative control miRNA (Fig. 7D,E). These results suggest the concept that exosomes derived from ethanol-treated hepatocytes horizontally transfer miRNA-122 to immune cells and modulate their function in pro-inflammatory cytokine production through affecting the HO-1 pathway.

### The pro-inflammatory effects of exosomes derived from ethanol-treated hepatocytes are prevented by exosome-mediated miRNA-122 RNAi delivery

To evaluate the therapeutic potential of our observations, we postulated that by introducing a miRNA-122 inhibitor to the recipient immune THP-1 cells, we can attenuate pro-inflammatory immune activation induced by exosomes derived from ethanol-treated hepatocytes (ethanol exosomes). We previously showed successful delivery of a miRNA inhibitor/mimic both *in vivo* and *in vitro* using an exosome-based delivery method^1^. Using the same methodology and exploiting THP1 derived exosomes as delivery vehicles, we loaded a miRNA-122 inhibitor or control inhibitor into the THP1 derived exosomes and treated THP1 cells with these “therapeutic” exosomes for 12 h. After, we washed off the “therapeutic” exosomes and exosomes derived from ethanol-treated Huh7.5 cells (ethanol exosomes) were added, along with groups containing control exosomes, LPS, and electroporated miRNA-122 as a positive control, and other control conditions. Our results showed that introducing miRNA-122 inhibitor “therapeutics” can attenuate the sensitization of THP1 cells to the LPS by exosomes from alcohol-exposed hepatocytes. This was demonstrated by decreased levels of TNFα production in THP-1 monocytes that received the “therapeutic” exosomes containing a miR-122 inhibitor prior to exposure to exosomes from ethanol-treated hepatocytes (p < 0.05) (Fig. 7F).

## Discussion

The protein and small RNA cargo of exosomes are unique and can be specific to the type and activation status of their parental cells. The biological significance of exosomes and their miRNA cargo in intercellular communication is yet to be fully understood^37^,^38^.

In our study, we present the novel finding that the number of exosomes is increased in the circulation after binge alcohol consumption in healthy human subjects and mice and after chronic alcohol feeding in mice. After isolating exosomes from healthy human subjects following binge alcohol drinking, we found elevated levels of miRNA-122 as early as 4 h after alcohol consumption that may indicate liver damage associated with acute alcohol binge. These data are in accordance with a previous study from our group, where we found elevated levels of miRNA-122 in the circulating exosomes of mouse models of alcoholic hepatitis (AH), drug (acetaminophen, APAP)-induced liver injury (DILI), and Toll-like receptor (TLR) 9 + 4 ligand-induced inflammatory cell-mediated liver injury^25^. Exosomes isolated from sera of ethanol-fed mice (5 weeks) and binge alcohol fed mice (12 h) contained significantly higher levels of miRNA-122 compared to the control mice.

Given that miRNA-122 is reported to be a liver specific miRNA^26^, we hypothesized that the source of exosomes that had elevated miRNA-122 were hepatocytes. To test this hypothesis, we evaluated the level of miRNA-122 in exosomes derived from Huh7.5 hepatocytes and primary human hepatocytes. Consistently, levels of miRNA-122 increased significantly after ethanol treatment, explaining the observed elevated levels of miRNA-122. Next, we asked whether these miRNA-122 harboring exosomes have downstream effects on monocytes. By treating THP1 monocytes with exosomes derived from ethanol-treated Huh7.5 cells, we showed that miRNA-122 which is indigenously almost absent in the monocytes, is horizontally transferred via exosomes from ethanol-treated hepatocytes to the THP1 human monocytes, and modifies their immune function (Supplementary Figure 1). These *in vitro* findings were corroborated by observation of increased miRNA-122 levels in inflammatory cells, Kupffer cells and liver mononuclear cells, isolated from chronic alcohol-fed mice.

Immunomodulatory activities including both immune-suppression and immune-activation, were reported for the exosomes. For example, exosomes isolated from culture supernatant of human neural stem cells (hNSC) were able to suppress the activation and proliferation of human T-cells by induction of apoptosis and G0/G1 cell cycle arrest^37^. In another study, exosomes secreted by B-cells were reported to stimulate CD4^+^T-cells in both murine and human B-cell lines^38^. Host macrophages infected with bacterial species like Mycobacterium, were found to secrete exosomes that induce pro-inflammatory response in uninfected cells through exposure to pathogen-associated molecular patterns, as an immune surveillance method^39^.

We found selective immune modulatory activity of exosomes derived from ethanol-treated hepatocytes to THP1 cells, and we showed that these exosomes sensitize THP1 cells to LPS stimulation and induce pro-inflammatory responses and over-expression of Nox2 through modulation of HO-1 expression. HO-1 has a reciprocal effect on Nox2 expression^36^, and it has been demonstrated that HO-1 expression in macrophages inhibited NADPH oxidase activity through decreased heme availability and Nox2 expression^40^. Together, our results indicate that alcohol sensitizes THP1 cells to LPS-induced pro-inflammatory activation by at least two mechanisms. First, by an activating effect on the exosome production machinery in hepatocytes indicated by increased number of miRNA-122 harboring exosomes and second, via miR-122 that can augment inflammation in immune cells. Exosomes derived from ethanol-treated Huh 7.5 cells were bioactive and bioavailable and were able to sensitize THP1 human monocytes to LPS and induce augmented inflammatory reactions compared to LPS and LPS + ethanol. These results add another facet to the previously observed pro-inflammatory phenotype in liver-resident and non-resident immune cells, suggesting that augmented pro-inflammatory cytokine production and reactive oxygen species generation may not only be due to alcohol and its metabolites, but also due to the paracrine activity of exosomes derived from hepatocytes after alcohol exposure.

Exosomes originate from secreted inter-luminal vesicles of multivesicular endosomes (MVEs). Exosome production and the transport system is controlled by the Rab family of small GTPases^41^. In the *in vivo* alcohol models and *in vitro* model of ethanol-treated Huh7.5 cells, we found elevated levels of exosomes after alcohol consumption and ethanol treatment, respectively, supporting that alcohol activated the exosome production machinery in the hepatocytes. We also found an elevated level of Rab 27b in the hepatocytes treated with ethanol. Rab 27b is found to play a role in the docking of MVEs at the plasma membrane^41^. Therefore, our results strengthen the link between effects of alcohol on MVEs and exosome production.

The observed increase in miRNA-122 expression in monocytes was a mere consequence of horizontal transfer of genetic material through exosomes, indicated by non-detectable levels of pri-miRNA-122 in human THP1 monocytes. Moreover, our data showed that horizontally transferred miRNA-122 is functionally active and could modify the function of monocytes through affecting the miRNA-122 target, HO-1, and augment monocytes inflammatory responses in the presence of LPS. The HO-1 signaling pathway is an important survival pathway essential for the maintenance and cell plasticity after LPS and reactive oxygen species (ROS) challenges^32^,^33^,^40^. Exposure of HO-1 deficient mice to endotoxin lead to increased cellular necrosis, increased pro-inflammatory response, and a higher mortality from LPS shock^42^,^43^. In this study we showed that the presence of horizontally transferred miRNA-122 hampered production of cytoprotective HO-1 in response to LPS in the monocytes. We have advanced the understanding of the role of transferred miRNA-122 and subsequent HO-1 pathway modulation by showing interplay between ethanol-treated Huh7.5 cells and THP1 human monocytes. Of note, we found that exosomes derived from non-alcohol treated Huh 7.5 cells can partially attenuate LPS-induced HO-1 expression. This is in line with other evidence reporting immune suppressive effects of exosomes originated from tumor cell lines, which is believed to be associated with several mechanisms including transportation of specific proteins (including Fas ligand and TRAIL) or immune suppressing miRNAs to immune cells^44^. We showed that exosomes derived from ethanol treated Huh7.5 cells transfer the mature form of miRNA-122 -liver specific miRNA- to THP1 cells, sensitizing them to LPS stimulus and inducing pro-inflammatory cytokines such as TNFα and IL-1β. To prove it was indeed the transferred miRNA-122 via the exosomes that induced pro-inflammatory cytokines, we conducted two independent “simulation experiments”, in which we overexpressed miRNA-122 in both THP1 cells and RAW macrophages. Results of both experiments revealed significantly increased pro-inflammatory cytokine production after miRNA-122 overexpression. Our results were consistent with what we observed in treatment of THP1 cells with exosomes derived from ethanol-treated hepatocytes and elevated levels of TNFα and IL-1β in the recipient cells.

Although the present study is the first report suggesting a role of miRNA-122 in pro-inflammatory pathophysiology of alcoholic hepatitis, elevated levels of miRNA-122 and subsequent increase in type 1 IFN production have been reported in hepatitis B^45^. In contrast to the fact that miRNA-122 is not normally expressed in T-cells, in cutaneous T cell lymphoma (CTCL) elevated levels of miRNA-122 were reported which were induced by p53 in response to chemotherapy. In T-cells, miRNA-122 was able to prevent apoptosis by stimulating the Akt kinase pathway^46^. This is in contrast to the pro-apoptotic activity of miRNA-122 in hepatocellular carcinoma and suggests that the role of miRNA-122 could be unique depending on the cell type.

In addition to the classical means of communication between cells through secretion of soluble factors, the cell-to-cell adhesion contact, and the intercellular exchange of organelles through nanotubular structures^47^, exosomes function as alternative intercellular communicators during disease states, and provide insights on developing therapeutic potentials. The use of an *in vitro* model of treatment of THP1 monocytes with hepatocyte-derived exosomes allowed us to demonstrate that the transfer of miRNA-122 through exosomes resulted in functional effects as documented by increased sensitivity of THP1 monocytes to LPS, to induce drastically more pro-inflammatory cytokines. Because exosome production is strongly increased in hepatocytes after ethanol administration, the blockage of functional effect of the miRNA-122 bearing exosomes could be a therapeutic target in AH. The present data provide, for the first time, the evidence of a specific mechanism through which hepatocytes communicate with monocytes by virtue of an active transfer of miRNA-122 into the target cells accomplished by exosomes. This mechanistic cross talk leads to sensitizing effects and augmented inflammatory responses in monocytes and can open up a whole new set of possibilities of therapeutic potential of miRNA-122 inhibitor as a preventive approach and treatment of alcoholic hepatitis. Pretreatment of monocytes with miRNA-122 inhibitor was able to prevent the pro-inflammatory induction activity of ethanol exosomes in THP1 monocytes. The targeted delivery of miRNA-122 inhibitor to the monocytes can be a possible therapeutic approach to attenuate alcohol-related inflammatory response.

In conclusion, our results revealed a novel mechanism of cross talk between ethanol-exposed hepatocytes and normal monocytes via exosomes and demonstrated that exosomes derived from ethanol treated hepatocytes can augment pro-inflammatory conditions and induce immune modulation in monocytes/macrophages characteristic of alcohol consumption. In addition to the well-documented alcohol and alcohol metabolites damages, immune modulation of exosomes through exosome-mediated transfer of miRNA-122 and suppression of the HO-1 pathway, could be an alternative mechanism of sensitization of monocytes/macrophages to LPS in AH. In this study we also showed that exosomes have therapeutic potential, and can be used as vehicles for gene and RNA interference delivery. We loaded exosomes with miRNA-122 inhibitor and used them as a therapy to attenuate miRNA-122 mediated immune stimulation in monocytes. In addition to adding novel mechanisms of cross talk of ethanol-treated hepatocytes with monocytes and its implication in AH, this study opened up new avenues in therapeutic potentials by using RNA interference in the recipient immune cells to ameliorate the detrimental effects of exosomes derived from alcohol-treated hepatocytes on monocytes.

## Materials and Methods

### Human Studies

Healthy male individuals (n = 11) with no history of alcoholism or alcohol use habits were enrolled in this study. The study was approved by Institutional Review Board for the Protection of Human Subjects in research at the University of Massachusetts Medical School. The methods were carried out in accordance with the approved guidelines. Written consents were obtained, files are kept in locked cabinets, and samples were de-identified. To qualify for the study, subjects had alcohol use of fewer than 12 drinks/wk^27^,^48^. Alcohol was given at 2 ml vodka 40% v/v ethanol/kg body weight in a total volume of 300 ml orange/strawberry juice in the Clinical Research Center (CRC). The control group took the same volume of orange/strawberry juice at the same time points. Blood was drawn at baseline and at 30 minutes, 1 h, 2 h, 3 h, 4 h, and 24 h time points post-alcohol consumption in serum separating tubes (BD Biosciences).

### Animal studies

The animal studies were approved, and conducted according to the regulations of the Institutional Animal Care and Use Committee (IACUC) of the University of Massachusetts Medical School (Worcester, MA, USA). Six to eight week old female *C57BL6/J* animals (n = 6 per group) were used for binge alcohol drinking and chronic alcohol feeding accompanied with appropriate controls. For chronic alcohol consumption model, the animals received 5% (v/v) ethanol (36% ethanol-derived calories) containing Lieber-DeCarli diet (EtOH) for 5 weeks and control animals received pair-fed diet (PF) with an identical amount of calories where the alcohol-derived calories were substituted with dextran-maltose (Bio-Serv, Frenchtown, NJ)^49^. The animals in the binge alcohol consumption group (n = 6) received 5 g/kg 50% (v/v) alcohol diluted in water via oral gavage. Animals were euthanized 6 h and 12 h after binge alcohol consumption. Blood was collected from animals and serum was separated from the whole blood.

### Kuppfer cell (KCs) and liver mononuclear cells (MNCs) isolation

KCs were isolated as described previously using an established protocol from pair-fed or alcohol-fed animals (n = 8)^50^. Briefly, after 10 minutes perfusion of liver with saline solution, *in vivo* digestion was done using liberase enzyme for 5 minutes and followed by *in vitro* digestion for 30 minutes. The non-hepatocyte farction was separated by Percoll gradient and centrifuged for 60 minutes at 800 × *g*. The inter-cushion fragment was washed and adhered to plastic in Dulbecco’s modified Eagle’s medium supplemented with 5% fetal bovine serum. The non-adherent fraction was washed, and the adherent KC population was lysed in QIAzol Lysis reagent (Qiagen, Maryland, USA) for subsequent RNA analysis. Liver mononuclear cells (MNCs) were isolated from another set of mice (n = 8) and separated by Percoll gradient using a previously described protocol^51^.

### Cell culture and exosome isolation

Huh 7.5 cells, primary human hepatocytes, and RAW 264.7 macrophages were cultured in Dulbecco’s modified medium (DMEM) plus 10% exosome-depleted FBS (Exo-FBS™) (Mountain View, CA, USA), and 1% penicillin/streptomycin (Gibco®, NY, USA). Primary human hepatocytes were obtained from the National Institutes of Health Liver Tissue cell distribution system. For ethanol treatments, 25 mM, 50 mM, and 100 mM of ethanol were added to the cells for various time points (24 h, 48 h, and 72 h). At desired time points, culture media was harvested and exosomes were isolated or quantified using a Nanoparticle Tracking Analysis (NTA) system. THP1 monocytes were cultured in RPMI media containing 10% exosome depleted FBS at 37 °C in a 5% CO_2_ atmosphere.

For exosome isolation from different sources of normal Huh7.5 cells, ethanol-treated Huh 7.5 cells, ethanol-treated primary human hepatocytes, normal primary human hepatocytes, and normal THP1 cells, supernatants were centrifuged at 1500 g for 10 minutes to remove cells followed by 10000 *g* for 20 minutes to deplete residual cellular debris. The supernatant was serially filtered through 0.8 μm, 0.44 μm and 0.2 μm. The filtered supernatant was used to precipitate exosomes with ExoQuick-TC™ (System Biosciences) (according to the manufacturer’s guidelines). After isolation, exosomes were re-suspended in PBS. For exosome isolation from sera of mice and human subjects, exosomes were isolated from 150 μl of sera with ExoQuick reagent (System Biosciences) following the recommended protocol. For co-culture experiments, exosomes isolated from Huh 7.5 cells (ethanol-treated or non-treated) were added to THP1 cells for 8 h in the concentration of 50–100 μg/ml. This concentration was comparable to exosome concentration in human subjects. After 8 h co-culture of exosomes with THP1 cells, cell culture suspension was transferred to the centrifuge tubes and THP1 cells were sedimented by centrifugation at 1000 RPM. 1000 RPM is just enough to sediment cells and based on density of exosomes they are unlikely to co-participate with the THP1 cells and they would be mostly on the supernatant and would be discarded. For ruling out any possible co-precipitation of exosomes with the cell pellet, cells were washed two times with PBS and centrifuged and harvested in the same manner. In the groups contained that LPS, 10 nM LPS was added 6 h before the readouts.

### Characterization of exosomes

Exosomes were characterized by Nanoparticle Tracking Analysis (NTA), transmission electron microscopy (TEM), and western blot in terms of size, concentration, morphology, and surface marker.

### Nanoparticle Tracking Analysis (NTA)

The concentration and diameter of exosomes derived from culture supernatant, sera of human subjects (n = 11), and mice were identified by a NanoSight NS300 system (NanoSight, Amesbury, UK) equipped with a fast video capture and Nanoparticle Tracking Analysis (NTA) software. The instrument was calibrated with 100 nm polystyrene beads (Thermo Scientific, Fremont, California, USA) before using. The samples were captured for 90 s at room temperature. NTA software was used to measure concentration of the particles (particles/ml) and size distribution (in nanometer). Each sample was measured three times.

### Transmission Electron microscopy

Isolated exosomes from Huh 7.5 cells were re-suspended in PBS and placed on a formvar-coated copper grid and then allowed to settle for 30 minutes. The grid was washed several times with PBS by positioning droplets of PBS on the top and applying absorbing paper in between. The specimen was fixed by placing the grid on the top of 2% paraformaldehyde placed on the parafilm for 10 minutes. Fixation was followed by several washes with deionized water and the sample was contrasted by adding 2% uranyl acetate for 15 minutes. The sample was embedded by adding a drop of 0.13% methyl cellulose and 0.4% uranyl acetate for 10 minutes. The grid was visualized using a Philips CM10 transmission electron microscope and images were captured using a Gatan CCD digital camera.

### Scanning Electron Microscopy (SEM)

Huh 7.5 cells were plated into 12-well plates. 50 mM ethanol was added to the ethanol group for 24 hours. Both control cells and ethanol-treated cells were fixed with a 2.5% glutaraldehyde in Sorenson Phosphate buffer (0.1 M, pH 7.4) for 2 h. After fixation, hepatocytes were washed three times in PBS (pH 7.4) for 5 minutes, and then fixed with 1% Osmium tetroxide in 0.1 M PBS (pH 7.4) for 1 h. Subsequently, samples were washed and dehydrated in a graded series of alcohol (30%, 50%, 75%, 85%, 95%, 100%). The procedure was followed by mounting the samples on a specimen stub and then sputter coating with gold/palladium. The samples were visualized with a MKII FEI Quanta 200 FEG MKII scanning electron microscope (FEI Company, Netherland).

### Western blotting

After isolating exosomes from Huh 7.5 cells, the presence of exosomal marker, CD63, was confirmed with western blot, using our laboratory’s established protocol. RIPA buffer was (Thermo scientific) added to the exosomes; exosomal proteins were extracted and run on 10% SDS-PAGE gels. Proteins were transferred to nitrocellulose membrane, and were blocked for 1 h in TBS containing 5% non-fat dry milk and 0.1% Tween-20. Afterwards, the blot was incubated overnight with primary CD63 antibody (Santa Cruz Biotechnology) at 4 °C. The blot was washed 3 times with TBST and then incubated for 1hour with horseradish peroxidase-conjugated secondary goat anti-mouse IgG-HRP antibody (Santa Cruz Biotechnology) (dilution 1:10,000). CD63 protein band was visualized on blot using a Clarity™ Western ECL substrate kit (BioRad) according to the manufacturer’s protocol and analyzed with a Fujifilm LAS-4000 luminescent image analyzer.

### Enzyme-linked Immunosobent Assay (ELISA)

Protein levels of MCP1, TNFα, and IL-1β were measured in cell free culture supernatant by ELISA. Levels of TNFα (BD Biosciences, San Diego, CA), MCP1 (BioLegend Inc., San Diego, CA) and IL-1β (R&D Systems, Inc., Minneapolis, MN) were measured based on manufacturer’s recommendations and quantified using an ELISA reader.

### Loading miRNA-122 inhibitor into the exosomes and THP1 pretreatment

We previously showed that exosomes can be used as successful delivery vehicles for functional delivery of RNA interferences *in vitro* and *in vivo*^1^. Using an optimized method for loading exosomes based on our previously optimized protocol, we loaded THP1 derived exosomes with miRNA-122 inhibitor and used them as vehicles to deliver miRNA-122 inhibitor to the naïve THP1 cells. Briefly, re-suspended exosomes that were diluted in Gene Pulser®electroporation buffer (Bio-Rad Laboratories, Berkeley, CA) in 1:1 ratio. MiRNA-122 inhibitor or negative control for miRNA inhibitor (Ambion, Grand Island, NY) at final amount of 300 pmol were added to the exosome sample containing 1 μg/μl exosomal protein. The mixtures were transferred into cold 0.2 cm electroporation cuvettes and electroporated at 150 kV and 100 μF. A Gene pulser II System (Bio-Rad Laboratories, Berkeley, CA) was used for electroporation. The exosomes were treated with one unit of RNase H to eliminate free-floating miRNA-122 inhibitor or negative control outside the exosomes and re-isolated using ExoQuick-TC™. These loaded exosomes were co-cultured with THP1 cells for 12 h. Afterwards, the THP1-derived loaded exosomes were washed off and exosomes derived from ethanol-treated Huh7.5 cells (ethanol exosomes) were added, along with groups containing control exosomes, LPS, direct electroporated miRNA-122 to the THP1 cells as a positive control, and other control conditions.

### Transfections via electroporation and transfection reagents

For direct introduction of miRNA-122 mimic (Ambion, Grand Island, NY) and HO-1 siRNA (Life Technologies) to the cells, THP1 cells were transfected by electroporation as follows: 2 × 10^5^ cells were re-suspended in 150 μl complete RPMI media and 150 μl Gene Pulser® electroporation buffer for 5 min on ice before being electroporated. Electroporation was done at 300 kV and 1500 μF. Following electroporation, cells were kept on ice for 10 min then cultured in media for 24 hrs. Scrambled control was introduced directly to THP1 cells using the same method.

For the introduction of miRNA-122 mimic to RAW macrophages, Lipofectamine® RNAiMAX (Life Technologies) was used based on manufacturer’s protocol for cell transfection. Cells were seeded 1 day before treatment and different treatment conditions, and controls including miRNA-122 mimic, negative control mimic, miRNA-122 with Lipofectamine*®* RNAiMAX, and miRNA-122 control mimic with, were applied for 48 hours. Afterwards, cells were washed and treated with 100 ng/ml LPS for 6 hours. Relative expression levels of HO-1 were measured by qPCR and TNFα protein levels were measured in supernatants by ELISA.

### Confocal Microscopy

Isolated exosomes from supernatant of Huh 7.5 cells were labeled with PKH67 green fluorescent cell linker kit (Sigma-Aldrich). Labeled exosomes were co-cultured with THP1 cells for 6 h and then washed off. After 8 h, an uptake of labeled exosomes by recipient THP1 cells was visualized by a Leica TCS SP5 II laser scanning confocal microscope equipped with a 63 × phase objective. Plasma membrane was contrasted with Nomarski interference microscope and nuclei were stained with DAPI (blue). Confocal stacked images (0.2 μm stack step, 1 μm range) were acquired and Imaris analytical software (Bitplane Scientific Software) was used to construct 3D projections of image stacks.

### RNA isolation

Both cells and exosomes were lysed in QIAzol Lysis reagent (Qiagen,Maryland, USA) and total RNA was isolated using Direct*-*zol*™* RNA MiniPrep isolation kit (Zymo Research Corp, Irvine, CA). Optical density (260/280 and 260/230 ratios) was measured to check RNA quality and quantity. 100 μL of exosome suspension from sera of patients after binge alcohol consumption, normal subjects, from supernatant of ethanol-treated cells, or normal cells were mixed with 500 μL QIAzol lysis buffer, and the mixture was processed according to the manufacturer’s standard protocol. The extracted RNA was eluted with 25–35 μL of RNase-free water. The quantity and quality of the RNA were determined by NanoDrop 1000 (260/280 and 260/230 ratios) for cells and Agilent Bioanalyzer 2100 with a Small RNA Chip for exosomal RNA.

### Quantitative real-time polymerase chain reaction (qPCR)

For mRNA analyses, cDNA was transcribed from 1 μg of total RNA utilizing iScript™ cDNA synthesis kit (Bio-Rad) in a final volume of 20 μl. SYBR-Green-based real-time quantitative PCR (qPCR) was performed using the iCycler (Bio-Rad Laboratories Inc., Hercules, CA) and the Bio-Rad CFX96 Real-time PCR Detection system (Bio-Rad Laboratories)^1^. The primer sequences were as follows: 18 S, forward, 5′-GACCTCATCCCA CCTCTCAG-3′, and reverse, 5′-CCATCCAATCGGTAGTAGCG-3′; human TNFα, forward, 5′- GAGTGACAAGCCTGT AGCCCATGTTGTAGCA -3′, and reverse, 5′- GCAATGATCCCAAAGTAGACCTGCCCA

GAC T -3′; human IL-1β, forward, 5′- CAGCTACGAATCTCCGACCAC-3′, and reverse, 5′- GGCAGGGAACCAGCATCTTC-3′; human MCP1, forward, 5′- CCCCAGTCACCTGCTGTT AT -3′, and reverse, 5′- TGGAATCCTGAACCCACTTC-3′; human Nox2, forward, 5′- GGGAAAAATAAAGGAATGCC -3′, and reverse, 5′- AGCCAGTGAGGTAGATGTTG-3′; human HO-1, forward, 5′- ACCAACTGCTTAGCACCC -3′, and reverse, 5′- GCAGAGAATGCTGAGTTCATG -3′; human GAPDH, forward, 5′-AGGGCTGCTTTTAACTCTGGT-3′; and reverse, 5′-CCCCACTTGATTTTGGAGGGA-3′; mouse HO-1, forward 5′-CTGTGTAACCTCTGCTGTTCC-3′, and reverse, 5′-CCACACTACCTGAGTCTACC-3′; human Rab 27b, forward, 5′- TGCGGGACAAGAGCGGTTCCG-3′, and reverse, 5′- GCCAGTTCCCGAGCTTGCCGTT-3′. mRNA levels were normalized against 18S (internal control) and relative levels were calculated using the ΔΔCt method. The relative expression level of each mRNA was presented by 2^–ΔΔCt^.

### miRNA analysis

TaqMan® miRNA Assays (Applied Biosystems, Foster City, CA) was used for detection of miRNA-122, miRNA-155, miRNA-29b expression according to manufacturer’s protocol, as described previously^1^. Reverse transcription (30 min, 16 °C; 30 min, 42 °C; 5 min 85 °C) was done using a TaqMan stem loop primer, 10 ng RNA, TaqMan primers and miRNA reverse transcription kit in a Eppendorf Realplex Mastercycler (Eppendorf, Westbury, NY). Quantitative real-time PCR was done in Bio-Rad CFX96 iCycler (Bio-Rad Laboratories) using TaqMan Universal PCR Master Mix. In primary human hepatocytes and Huh 7.5 cells, RNU-48 was used to normalize the Ct values between the samples. snoRNA202 was used to normalize Ct value in liver mononuclear cells and Kupffer cells. In experiments involving miRNA analysis of exosomes, synthetic C. elegans (cel)-miRNA-39 was spiked during the total RNA isolation process and used to normalize the qPCR data. TaqMan® Pri-miRNA Assays were done using FAM dye- labeled TaqMan with GAPDH as internal control. For each sample, two independent reverse transcription reactions were done, and each experiment was done in triplicate. miRNA levels were normalized, and the relative expression levels of specific miRNA were presented by 2^–ΔΔCt^.

### Statistical Analysis

Based on data distribution, one-way analyses of variance (ANOVA) or Kruskal-Wallis nonparametric test were used to compare different groups. Student’s *t* test or Mann-Whitney *U* test were performed for comparing two groups. Data are presented as mean ± standard error of mean (SEM). P values less than 0.05 was considered as statistically significant.

## Additional Information

**How to cite this article**: Momen-Heravi, F. *et al*. Exosomes derived from alcohol-treated hepatocytes horizontally transfer liver specific miRNA-122 and sensitize monocytes to LPS. *Sci. Rep.* **5**, 9991; doi: 10.1038/srep09991 (2015).

## Supplementary Material

Supplementary Information
