# Supplementary material for: Exosomes derived from alcohol-treated hepatocytes horizontally transfer liver specific miRNA-122 and sensitize monocytes to LPS
Source: Sci Rep. 2015 May 14;5:9991. doi: 10.1038/srep09991 (PMC4650752; doi:10.1038/srep09991)
Supplement: Supplementary Information [file srep09991-s1.pdf]

Supplementary Figure

Title: Exosomes derived from alcohol-treated hepatocytes horizontally transfer liver specific miRNA-122 and sensitize monocytes to LPS

Authors: Fatemeh Momen-Heravi*, Shashi Bala*, Karen Kodys, Gyongyi Szabo

*These authors contributed equally to this work.

[1]Department of Medicine, University of Massachusetts Medical School, Worcester, MA 01605, USA.

Corresponding Author: Gyongyi Szabo, MD PhD

Supplementary Figure 1

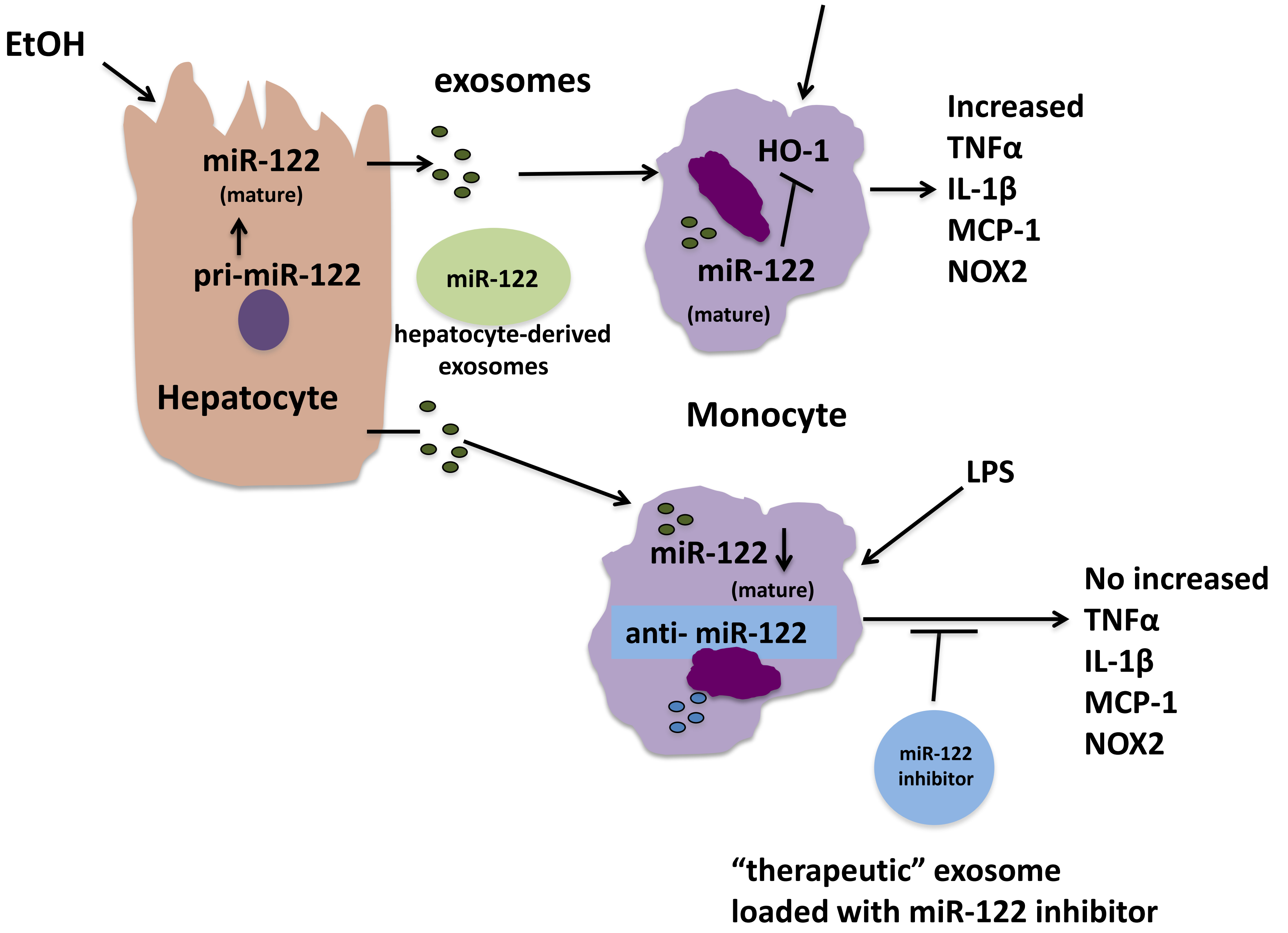

Supplementary Figure 1- This schematic depicts the experimental design and activated pathway after treatment of THP1 human monocytes with exosomes derived from ethanol-treated Huh7.5 cells.
